# 

**Published:** 1903-01-15

**Authors:** 


### SUBJECT INDEX TO VOLUME LVII.

### ADDRESSES AND PAPERS.

Army Dental Surgeon and his Work in the Philippines, G. D. Boak, 301.
Annealing Gold, J. R. Callahan, 109.
Anodyne Remedies, N. S. Hoff, 351.
Are the Teeth Deteriorating more Now than in the Past? F. H. Essig, 359.
Benefits of Dental Societies, W. A. Barber, 561.
Clinics at the Ohio State Meeting, 163.
Dental Instruction in the Public Schools, 217.
Dental Embryology, G. S. Blanchard, 1.
Easy Methods of Starting Gold Fillings, 169.
First Molar Extractions During Childhood and its Effects, M. F. Watson, 547.
Fitting a Davis or Logan Crown, A. F. Miller, 163.
Function and Value of the Dental Society, N. S. Hoff, 171.
Gold Crowns, H. E. Jenkins, 165.
Inlays for Difficult Cavities, J. S. McCurdy, 165.
International Dental Federation, H. C. Smith, 81.
International Dental Federation, 565.
Matrices, their Use and Retention, L. P. Hall, 399.
Medicated Gutta-Percha Points, L. T. Canfield, 167.
Modern Use of Soft Foil, G. S. Jungerman, 10.
New Method of Soldering, F. W. Stephan, 164.
New Porcelain Crown, J. P. Carmichael, 513.
Obtunding Sensitive Dentine with Cataphoresis, W. A. Price, 83.
Our Dental Law, H. K. Lathrop, 433
Practical Methods in Porcelain Filling, H. C. Raymond, 288.
Practical Points in Practice, C. C. Noble, 604.
Predisposing Factors in Dental Caries, E. C. Kirk, 55.
President's Address, E. A. Honey, 508.
President's Address, Otto Arnold, 29.
President's Address, C. H. Worboys, 225.
Proceedings of Southwestern Michigan Society, 225.
Porcelain as a Filling Material, H. C. Raymond, 379.
Porcelain, O. N. Lanphear, 234.
Radical Cavity Preparation, H. M. Seemans, 17.
Relations of the Board of Examiners to the Profession, H. C. Brown, 73.
Replanting Teeth, G. S. Hadley, 228.
Some Phases of Pyorrhea and its Treatment, G. W. Cook, 487.
Some Pathologic Lesions of the Pulp and How to Treat Them, G. W. Cook, 283.
Taft, Jonathan, N. S. Hoff, 541.
Teeth in Relation to Degeneracy, H. C. Rutter, 36.
Variations in Development and Density of Tooth Structure, J. Taft, 271.
What Shall we do about Porcelain? E. A. Honey, 595.

### REPRINTS.

Adrenalin and Cocaine, M. Lermoyez, 255.
Bleaching Enamel, J. Head, 419.
Cocaine Poisoning, a Case of, H. Prinz, 310.
Cavity Linings, B. L. Thorpe, 620.
Compressed Air in Dentistry, E. K. Knowles, 135.

Conservative Treatment of the Antrum of Highmore, Preston M. Hickey, 627.
Construction of a Matrix, G. W. Dittmar, 253.
Dental Curriculum of Sweden, L. Lindstroem, 97.
Dental Education, C. N. Johnson, 179.
Dental Germicides, E. A. MaWhinney, 408.
Dental Legislation, J. A. McGeough, 127.
Dental Law for Philippines Island, 207.
Diet for Pyorrhoea Alveolaris, F. W. Achorn, 501.
Echafolta, B. F. Thielen, 462.
English Dental Degree The, 245.
Ethics, E. A. Boyne, 623.
Failure of Dental Operations, F. L. Platt, 238.
Fillings as Barriers to Bacteria, A. E. Webster, 525.
Formaldehyde in Root Canals, B. F. Thielen, 526.
Gutta-Percha for Crown Setting, H. Williamson, 164.
Gutta-Percha Cement, E. Herbst, 313.
Immediate Root Filling, O. E. Inglis, 570.
Italian Dental Curriculum, V. Guerini, 187.
Lower Partial Plates, John Bauer, 460.
Manipulation and Care of Cements, W. V-B. Ames, 190.
Method of Taking Impressions, 312.
More Knowledge of Dental Text Books, A. E. Webster, 196.
New Local Anesthetic, 151.
Non-Cohesive Gold Fillings, G. W. Haskin, 250.
Our Professional Future, C. A. Van Duzee, 449.
Pay No License Fees, J. N. Crouse, 468.
Preliminary Education of a Dentist, C. M. Wright, 87.
Preliminary Education and College Training, M. Roy, 90.
Preliminary Examination of the Teeth, E. A. Bogue, 194.
Preventive Dentistry, C. M. Wright, 456.
Professional Ethics, C. N. Johnson, 421.
Professional Spirit, C. N. Johnson, 198.
Pyorrhoea Alveolaris, E. F. Wetzel, 242.
Preparing the Mouth for Anesthesia, Prof. Friteau, 254.
Removal of the Pulp Painlessly, W. C. Davis, 365.
Right to Administer Drugs in Dental Practice, 523.
Root Canal Fillings, J. I. Hart, 325.
Root Canal Fillings, B. F, Arrington, 148.
Some Historic and Chemical Facts Relating to the Welding of Gold, H. W. Morgan, 441.
Some Points in the Correction of Irregularities, R. Markwell, 415.
Some Unappreciated Causes of Anemia in Childhood, W. C. Hollopeter, 520.
Staple Crown, F. L. Marshall, 568.
Taft, Dr. J., Press Comments on Life, 575.
The Lower Denture, R. C. Brophy, 610.
University Degree for English Dentists, Wm. Guy, 339.
Welshers, H. McManus, 517.
Winning the Confidence of Children, E. P. Wentworth, 143.
X-Ray Vision, J. A. Smalley, 115.

### EDITORIAL.

American Society of Orthodontists, 582.
"A Good Rule," (?), 257.
Beauman, J. B. 100.
Broader Education for Scotch Dentists, 367.
California's New Dental Law, 258.
Central Michigan Dental Association, 157.
College vs. State Board Standards, 428.

Comment on Year's Events, 628.
Dental Cosmos, 101.
Dental Register, 46.
Dental Summary, 102.
Dominion Dental Journal, 102.
Duty of the 2,000 Newly Graduated Dentists, 473.
Harvard Dental College and the Four Year Course, 154.
Illinois New Law Vetoed, 314.
Illinois Dental Law, 153.
Improvement in Filling Teeth 258.
Institute of Dental Pedagogics, 48, 582.
International Dental Journal, 102.
Items of Interest, 102.
List of College Graduates for the Year, 474.
Master of Dental Surgery Degree in New York, 100.
Michigan State Dental Association, 423.
National Meetings at Asheville, N. C., 471.
New College Year, 470.
Odontographic Society of Chicago, 48, 153.
Ohio State Dental Society, 47.
Regeneration of the Pulp, 155.
Taft, Dr. J., 581.
Texas Dental Journal, 100.
Wisconsin's New Dental Law, 424.

### ANNOUNCEMENTS.

American Society of Orthodontists, 584.
Barrett, Dr. W. C., 156.
Bethel, Dr. L. P., 260.
Bodecker, Dr. H. C., 210.
Central Michigan Society, 316.
Chicago Dental Society, 315.
Columbus Dental Society, 305.
Dentists Pay City License, 315.
Detroit Dental Society, 261, 316.
Detroit Dental College Alumni Association, 476.
Eastern Indiana Dental Society, 317.
Fraternal Dental Society of St. Louis, 530.
Fraudulent Degree Holders Punished in Germany, 260.
Illinois State Society, 316.
Indiana Board of Examiners, 259.
Indiana State Dental Society, 209.
Institute of Dental Pedagogics, 157, 530.
International Dental Congress, 465, 631.
Jackson Michigan Dental Society, 315.
Kentucky State Dental Society, 260.
Kirk, Dr. E. C. Honored, 156.
Louisville College Alumni Association, 156.
Michigan State Dental Association 210, 318, 423.
Michigan Board of Examiners, 209, 259.
Missouri State Association, 210, 260, 368.
Missouri State Examiners, 316.
Muskegon Society Officers, 315.
National Dental Association, 317, 363.
National Association of Examiners, 530, 531.
National Association of Faculties, 317.
National Meeting at Asheville, 368
New York 7th and 8th District Societies, 530.
Northern Ohio Dental Society, 261
Odontographic Meeting Picture, 209.
Odontological Society of Western Pennsylvania, 106.
Ohio State Society, 106, 584, 585.
Ohio Board of Examiners, 261, 530.
Ohio Medical University Graduates, 259.
Pennsylvania Board of Examiners, 260.
Peoria Dental Society, 316.
Philadelphia Dental College Anniversity, 631.
Resignation of Professors, 315.
Southwestern Michigan Dental Association, 157.
Stockton, Dr. C. S., Dinner, 156.
Stra th, Dr. S., 259.
Toledo Dental Society, 318.
West Virginia Board of Examiners 209, 259.

Whitslar and Wilson, Removal, 106

### BIBLIOGRAPHY.

Long's Dental Materia Medica, 208.
Medical Critic, 211.
Medical Epitome Series, 538, 634.
Porcelain Inlays, 157.
Regional Anatomy, 369.
Standard Dictionary, 630.
Syphilis in Dentistry, 370.

### OBITUARY.

Otto Arnold, 262.
W. C. Barrett, 531.
Samuel T. Jones, 211.
J. B. Kirk, 263.
Herbert Lehr, 533.
S. B. Palmer, 263.
Jonathan Taft, 586.

### INDENTS.

A Business Method, 636.
Adhesive Aids in Starting Gold Fillings, 323.
Adrenalin as a Hemostatic, 322.
Adrenalin as a Resuscitant, 319.
Alopecia of the Mustach, 376.
Amalgam Filling, 264, 486.
Amalgam Mixer, 590.
Anchoring Lower Plates, 372.
Annealing, 477.
Anesthesia, Local, 477.
Antidolorin as a General Anesthetic, 538.
Antidolorin for Diagnosis, 590.
Antidolorin as a Local Anesthetic, 590.
Antidolorin in Minor Surgery, 534.
Antidote for Phenol, 266.
Artificial Dentures, 430.
Babbitt Metal, 159.
Bad Breath, 159.
Blackmail, 322.
Bleaching Teeth, 160.
Bridge Work, 429, 484.
Bridge Work a Compromise, 480.
Broader Culture, 480.
Bromo-Chloron, 427.
Canal Points, 588.
Carborundum Points, 372.
Care of the Hands, 537.
Cause of Vulcanite Breaking, 268.
Cavity Varnish, 269.
Celluloid Lacquer, 479.
Chemical Test for Sodium Dioxiode, 477.
Children's Teeth to be Cared For, 591.
Chinese Dentistry, 535.
Chloroform Liniment, 536.
Chronic Alveolar Abscess, 265.
Cleaning Broaches, etc., 265.
Cleaning Cement Slabs, 371, 478, 591.
Cleaning Cement Spatulas, 588.
Cleaning Trays, 264.
Cleaning Vulcanite Files, 476.
Cleaning Molten Zinc, 319.
Cocaine Solution for Pyorrhoea Work, 266.
Conduct of Colleges, 478.
Courtesies to Physicians, 159.
Damage Suits, 322, 483.
Dental Vestibules, 539.
Making a Dentist, 428.
Dont's, 431.
Draining Cavities of Thick Pus, 478.
Duplicating Vulcanite Dentures, 324.
Electric Furnace Lining, 588.
Electric Ozonation, 108.
Electricity in the Dental Office, 534.
Equivocal Comment, 482.
Erasing Powder, 264.
Ether Anesthesia Points, 371.
Eugenol for Sensitive Dentine, 478
Evacuating Pus, 264.
Extracting Roots Without Injuring the Process, 268.
Factors of Success, 482.
Fastening Teeth to Wax Base Plate, 476
Fatalities, 52.
Fever Blisters, 107.
Finishing Rubber Plates, 476.
Fixing Points in Socket Points, 160.
Formaldehyde Antidote, 477.
Formaldehyde for Canal Cleansing 535.
Formaldehyde as a Disinfectant, 264.

Foreigners as Technickers, 626.
Fruit Preservor, 479.
Fundamentals of Artificial Dentures, 591.
Fusing Points of Porcelain, 213.
Gold Crowns, How to make, 636,
Guaiacol in Pulpitis, 477.
Handy Soldering Block, 592.
Heterogenesis, 432.
Hint on Root Filling, 371.
Hydronaphthol in Cavity Lining, 373.
Illegal Practitioners, 485.
Improved Rubber Cups for Polishing Teeth, 107.
Increased Standard of State Examinations, 425.
Insanity from Nitrous Oxide, 589.
Inserting Gutta-Percha Points, 590.
Inserting Gutta-Percha Cement, 212.
Instruments, Acquaintance With, 483.
Intra-Venous Injection of Formaldehyde, 215.
Investment for Inlany Work, 265.
Investment Material, 594.
Listerine to Preserve Hydrogen Dioxide, 481.
Literature in Offices, 540.
Lockjaw from Decayed Teeth, 161
Logan Crowns, Adapting, 634
Mending Plaster Teeth, 589.
Mentho-Phenol, 266.
More Technic Work in Colleges, 425.
Mouth Breathing and Respiratory Obstructions, 536.
Mouth Wash, 254.
Napkin Point, 213.
Neuralgia, 319, 478.
Neuralgia Lotion, 319.
Neutral Soap, 634,
New Obtundent, 319.
Obtunding Dentine, 321.
Obtunding a Tooth for Crown Preparation, 635,
Odors in Dental Offices, 374.
Osmic Acid for Exposed Pulps, 479
Orthodontia, 592.
Oxyacetylene Blowpipe, 320.
Oxyphosphate for Inlay Impressions, 265.
Perfectly Fitted Clasps, 50.
Pepsin Pulp Digester, 160.
Phenol, Mono-Chloride, 483.
Plaster Bench, 589.
Physical Principles in Dentistry, 270.
Plate Making, 484.
Platinum Solder, 375.
Poisoning from External Use of Chromic Acid, 265.
Poisoning from Powerful Antiseptics, 594.
Poisoning from Supra-Renal Solutions, 374.
Polishing Proximal Surfaces, 159.
Polishing Gold Fillings, 588.
Polishing Vulcanite Gum Festoons 159.
Polishing Stains from Lower Incisors, 425.
Porcelain, Fusion, Strength and Shrinkage, 637.
Position of the Molar Teeth, 267.
Practices not Transferable in Germany, 591.
Pressure Anesthesia, 589.
Preventing Rust in Hypodermic Needles, 477.
Preventing Plaster Adhering to Flasks, 589.
Preventing Mellotts Metal Becoming Brittle, 372.
Protecting Cement Fillings from Moisture, 480.
Proprietarv Medicines, 485.
Pulp Canal Filling Material, 251.
Pulpless Teeth and Neurosis, 214.
Pulp Devitalization, 376.
Pulp Extirpation, 265, 267.
Purifying Wax, 377, 588.
Pyorrhea, A Case, 637
Refitting Lower Vulcanite Plates, 321.
Removing Posts Cemented into Roots, 107.
Removing Pulp, 593.
Removing Silver Stains, 267.
Replacing Broken Crown, 264.
Re-Soldering, 476.
Restoring Broken Incisor, 373.
Retaining Splint, 593.
Root Filling Material, 635.
Roughened Base of Inlay, 480.
Sensitive Buccal Cavities, 107.

Sensitive Roots, 159.
Sensitive Dentine, 588, 589.
Separating from Amalgam Filling, 592.
Separating Medium, 589.
Septoforma, 481.
Setting a Band Crown Painlessly, 108.
Should Colleges Educate Scientists? 214.
Shrinkage of Plaster Prevented, 588.
Soda for Sterilizing Canals, 592.
Soda for Toothache, 476.
Soldering Fluid for Gold, 160.
Somnoform, 320.
Statistics of Ethyl Chloride, 375.
Sterilizing Broaches, 212.
Sterilizing Instruments, 593.
Stopping Hemorrhage, 371.
Supports for Baking Crowns, 590.
System in the Dental Office, 50.
Third Molar, Removal of Impacted, 638.
Thorough Malleting of Contact Points, 269.
Thumb Sucking Case, 479.
Tooth Brushes, 53.
Tooth Corner Restored, 268.
Two Teeth for One Drunk, 167.
Value of a Tooth, 320.
Voice Lost with Tooth Extraction, 476.
Waxing up; Artificial Teeth Made Easy, 267.
What Should be Taught in the Fourth Year, 378.
Wine of Opium, 266.
Wisconsin Dental Law, 426.

AUTOGRAPHIC.

Achorn, F. W., 501.
Albright, W. H., 372.
Ambler, H. L., 41, 115.
Ames, W. V-B., 190.
Arnold, Otto, 29.
Arrington, B. F., 148.
Austin, Dr., 589.
Barbor, W. A., 561.
Baker, Dr., 375.
Barker, D. W., 428.
Barrett, W. C., 156.
Bartlett, W. M., 617, 619.
Bauer, John, 460.
Beauman, J. B., 27.
Black, G. V., 429.
Blanchard, G. S., 1.
Boak, G. D., 301.
Bodecker, H. C., 210.
Bodecker, C. F. W., 266
Bogue, E. A., 194, 625.
Brewster, R., 397.
Bridges, J. S., 480.
Brophy, T. W., 70, 156.
Brophy, R. C., 611.
Brownlee, W. A., 377, 589.
Brown, H. C., 73.
Bruck, W. H., 157.
Bryant, E. A., 430.
Buckley, J. P., 372, 477.
Bush, W. N., 377.
Callahan, J. R., 109, 115.
Callahan, P. H., 479.
Cameron, A. C., 107.
Campbell, W. D., 231.
Canfield, L. T., 167.
Capon, W. A., 213.
Carmichael, J. P., 513.
Carpenter, E. R., 373.
Chadwick, C. B., 232.
Chambers, Dr., 482.
Chisholm, G., 592.
Chittenden, C. C., 478.
Clark, H., 264.
Cochran, B. L., 635.
Cook, E. M., 15, 231, 232, 237, 281, 283, 487, 500.
Crouch, F. G., 269.
Crouse, J. N., 468.
Cruzen, E. E., 264.
Cudworth, W. H., 392.
Curtis, G. L., 108.
Curtis, Lenox, 639.
Custer, L. E., 28, 114.
Dailey, Dr., 333.
Davis, W. C., 365.
Deichmiller, 139.
Dieterich, M. P., 634.
Dittmar, 253.
Dodel, X., 588.
Edwards, L. D., 371.
Eubank, A., 265.
Essig, F. H., 359.
Evans, G., 329, 337.
Ezard, H. B., 346.
Fall Delos, 217.
Fletcher, M. H., 25, 70.

Fowler, S. M., 237
Friteau, Prof., 254.
Goslee, H. J., 476.
Green, M., 537.
Greevert, Dr., 268.
Guerini, V., 187.
Guilford, S. H., 324.
Guy, Wm., 339, 351.
Hadley, G. S., 228, 233.
Hall, L. P., 399.
Hanning, Dr., 333.
Harlan, A. W., 156.
Hart, J. I., 325, 327, 338.
Herzog, J. R., 589.
Harvey, H. F., 115.
Haskins, G. W., 250.
Hatch, Dr., 327, 328.
Hathaway, E. E., 609.
Hawley, C. B., 28.
Head, J., 419.
Heckard, W. A., 479.
Herbst, E., 313.
Hersh, W. H., 265.
Hewitt, Dr., 484.
Hogarth, B. W., 371.
Hogey, J. W., 593.
Hoff, N. S., 171, 351, 499, 601, 610.
Holopeter, W. C., 520.
Honey, E. A., 508, 595, 603.
Hooper, N. E., 230.
Howe, J. M., 266.
Hunt, G. E., 213, 534.
Hungerford, C. L., 480, 483, 537, 619.
Inglis, O. E., 570.
Ivy, R. S., 535.
Jack, L. F., 268.
Jelly, H. A., 594.
Jenkins, Chas. W., 157.
Jenkins, H. E., 165.
Johnson, C. N., 159, 179, 198, 269 421.
Junkerman, G. S., 10, 16.
Kirk, E. C., 55, 72, 114, 156.
Knowles, S. E., 135.
Lange, Dr., 322.
Lanphear, O. E., 160, 234.
Lathrop, H. K., 433.
Laville, Dr., 324.
LeCron, D. O. M., 639.
Lermoyez, M., 255.
Leonard, N. C., 270, 588.
Lindstroem, L., 97.
Lindsley, D. C., 348, 620.
Lodge, E. B., 499, 531.
Logan, J. D., 349.
Long, E. H., 208.
McCardie, W. J., 375.
McCurdy, J. S., 165.
McFarlane, Dr., 482, 539.
McGeough, J. A., 127.
McManus, H., 517.
Magee, J. M., 590.
Markwell, R., 415.
Marshall, F. L., 568.
Marshall, Dr., 486.
Martin, O., 637.
MaWhinney, 408, 638.
Mead, B. E., 320.
Miller, A. F., 163.
Mills, W. A., 159.
Morgan, H. W., 441.
Moore, E. C., 397,
Moorman, H. M., 603.
Morse, R. W., 282.
Noble, C. C., 604.
Noyes, F. B., 432.
Noyes, E., 378.
Oakman, C. H., 396, 497.
Palin, J. H., 232, 281.
Palmer, G. L., 592.
Patterson, J. D., 634.
Peak, O. L., 107.
Pepperling, T. L., 108.
Perry, S. B., 333, 334.
Platt, F. S., 238.
Post, C. E., 588.
Pratt, H. L., 477.
Price, W. A., 83.
Prinz, H., 310, 619.
Prosser, Dr., 425.
Prothers, J. H., 590.
Raymond, H. C., 288, 378, 379.
Reid, J. G., 531.
Rhein, M. L., 215, 329.
Roberts-Austin, W. C., 477.
Robinson, J. A., 267.
Roe, C. B., 232.
Rose, Harry, 268.
Roy, Maurice, 90.
Ruggles, S. D., 26.
Russell, J W., 538.
Rutler, H. C., 36.
Semans, H. M., 17.
Sheehan, D. E., 169.
Shiach, J. G., 346.
Smalley, J. A., 115.
Smith, A. G., 594.

Smith, F. H., 159.
Smith, H. T., 114.
Smith, H. A., 67.
St. Clair, C. W., 373.
Stephen, J. A., 16, 29, 113.
Stephan, F. W., 164.
Stevens, B. Q., 618.
Stockton, C. S., 156.
Stubblefield, Dr., 481.
Schaeffer-Stuckert, Dr., 151.
Taft, J., 223, 238, 271, 282, 476, 541.
Taggart, W. H., 373, 476.
Talbot, E. S., 42, 71.
Thielen, B. F., 462, 526.
Thompson, J. M., 235, 237, 282, 321, 395, 590.
Thorton, A. W., 588, 589.
Thorbe, B. L., 531, 621.
Todd, W. H., 113, 593.
Truman, W. H., 214, 536.
Tuller, R. B., 320.
Turner, P. S., 371.
Turnbull, F. T., 347.
Van Duzee, C. A., 449.
Van Orden, Dr., 588
Van Woert, F. S., 265.
Wallace, J. F., 617.
Warren, G. E.,
Watson, M. T., 457.
Watts, J. R., 264.
Watt, J. N., 350.
Webster, A, E., 525.
Weiss, O. A., 591.
Weld, F. A., 476.
Wentworth, E. P., 143.
Wessels, Dr., 270.
Wetzel, E. F., 242.
Wheat, W. H., 476.
Whitslar, W. H., 106.
Williamson, H., 164.
Williamson, Dr., 344.
Willmot, J. B., 426, 431, 615.
Winter, G. B., 212.
Wilson, Geo. H., 106.
Worboys, C. H., 237, 396, 609.
Wright, C. M., 72, 87, 456,
Young, J. L., 635
Zederbaum, G., 371, 591.

